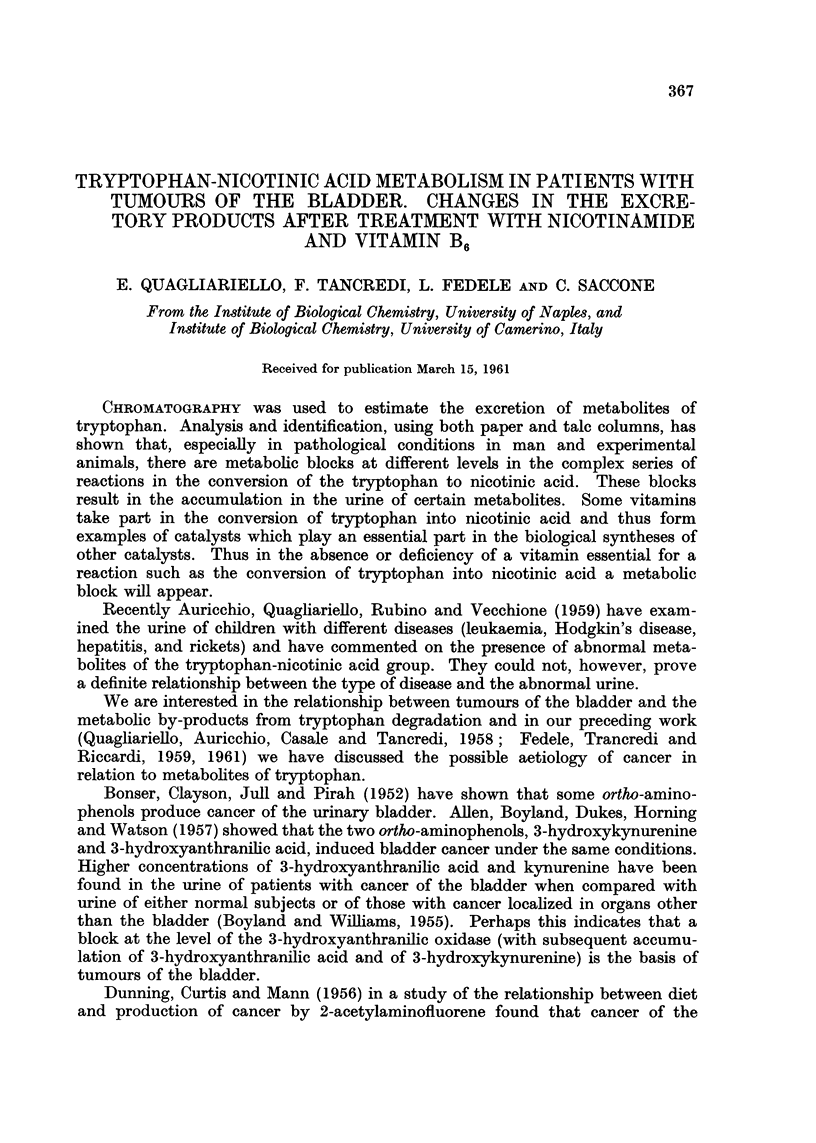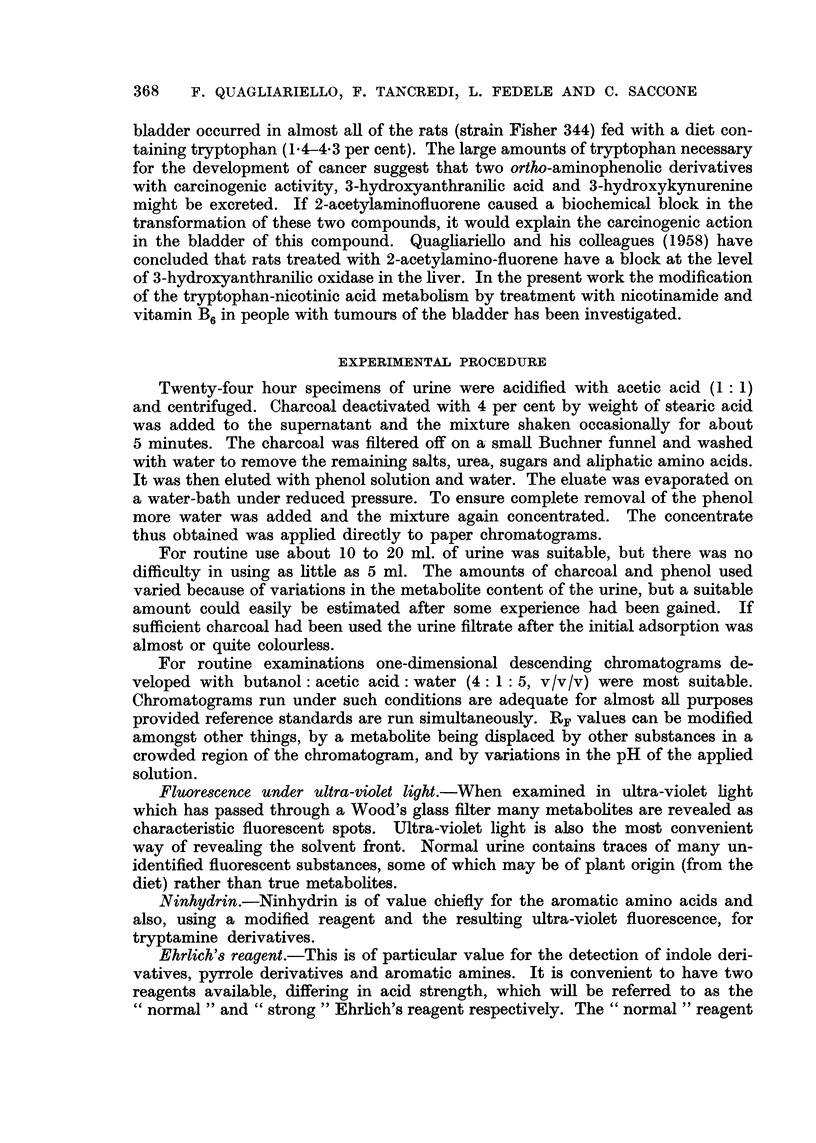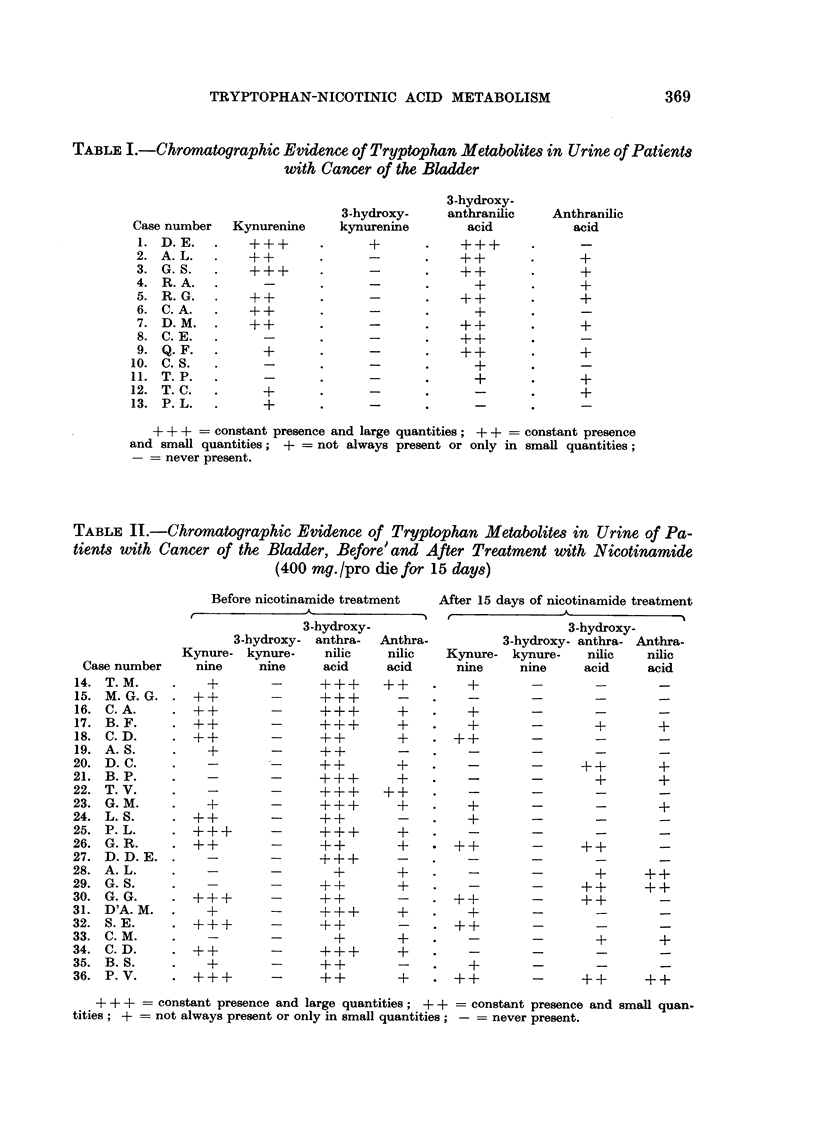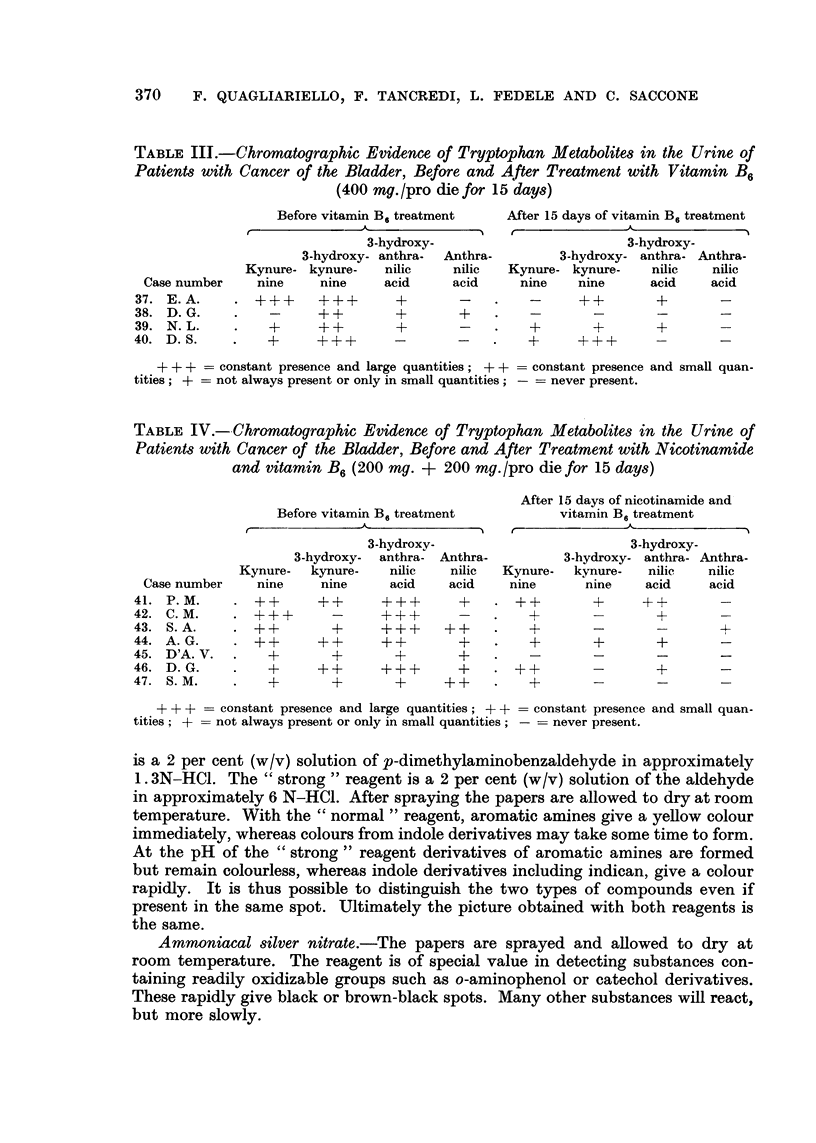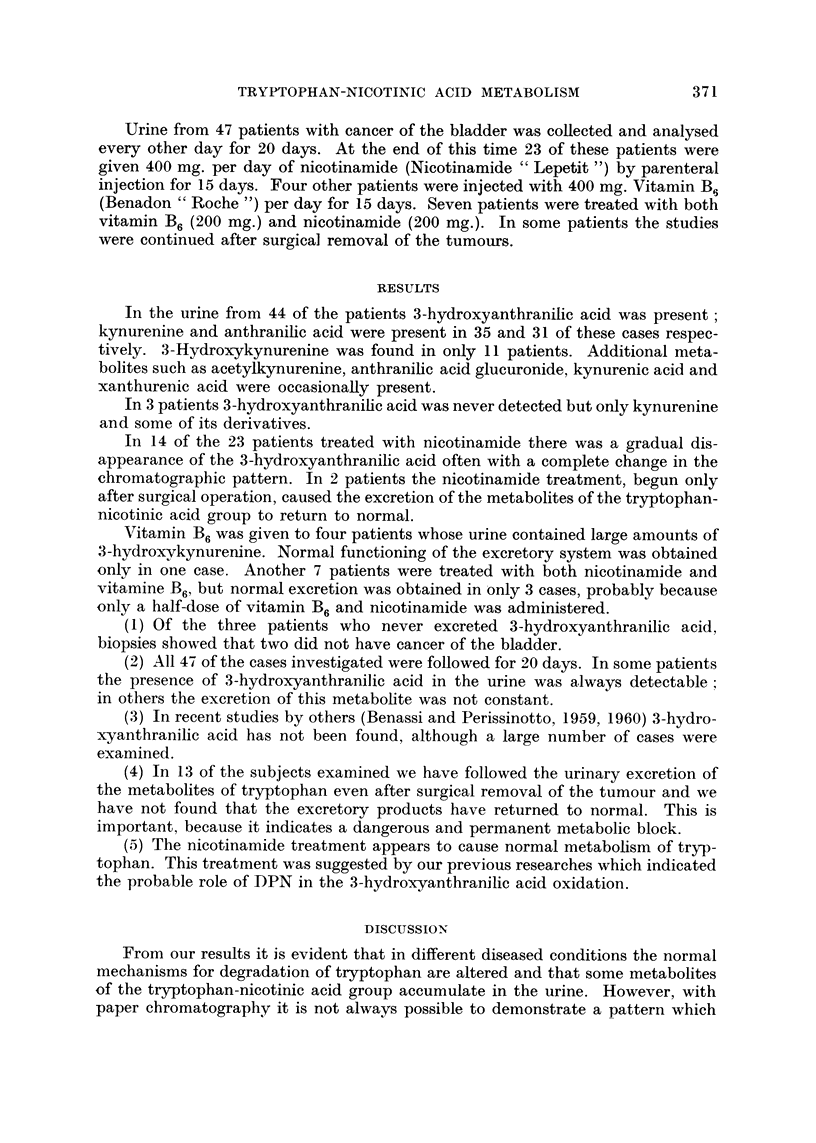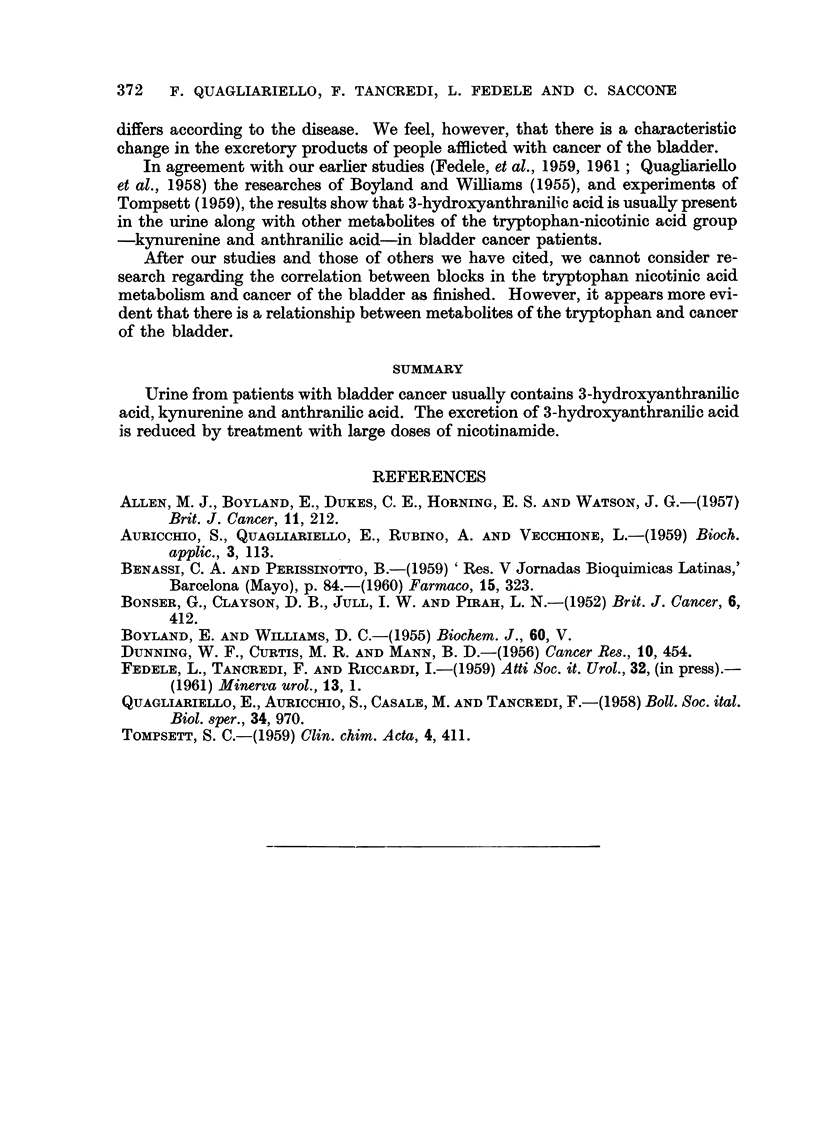# Tryptophan-nicotinic Acid Metabolism in Patients with Tumours of the Bladder. Changes in the Excretory Products after Treatment with Nicotinamide and Vitamin B_6_

**DOI:** 10.1038/bjc.1961.47

**Published:** 1961-06

**Authors:** E. Quagliariello, F. Tancredi, L. Fedele, C. Saccone


### TRYPTOPHAN-NICOTINIC ACID METABOLISM IN PATIENTS WITH TUMOURS OF THE BLADDER. CHANGES IN THE EXCRETORY PRODUCTS AFTER TREATMENT WITH NICOTINAMIDE AND VITAMIN $B_6$

E. QUAGLIARIELLO, F. TANCREDI, L. FEDELE AND C. SACCONE

*From the Institute of Biological Chemistry, University of Naples, and Institute of Biological Chemistry, University of Camerino, Italy*

Received for publication March 15, 1961

CHROMATOGRAPHY was used to estimate the excretion of metabolites of tryptophan. Analysis and identification, using both paper and talc columns, has shown that, especially in pathological conditions in man and experimental animals, there are metabolic blocks at different levels in the complex series of reactions in the conversion of the tryptophan to nicotinic acid. These blocks result in the accumulation in the urine of certain metabolites. Some vitamins take part in the conversion of tryptophan into nicotinic acid and thus form examples of catalysts which play an essential part in the biological syntheses of other catalysts. Thus in the absence or deficiency of a vitamin essential for a reaction such as the conversion of tryptophan into nicotinic acid a metabolic block will appear.

Recently Auricchio, Quagliariello, Rubino and Vecchione (1959) have examined the urine of children with different diseases (leukaemia, Hodgkin's disease, hepatitis, and rickets) and have commented on the presence of abnormal metabolites of the tryptophan-nicotinic acid group. They could not, however, prove a definite relationship between the type of disease and the abnormal urine.

We are interested in the relationship between tumours of the bladder and the metabolic by-products from tryptophan degradation and in our preceding work (Quagliariello, Auricchio, Casale and Tancredi, 1958; Fedele, Trancredi and Riccardi, 1959, 1961) we have discussed the possible aetiology of cancer in relation to metabolites of tryptophan.

Bonser, Clayson, Jull and Pirah (1952) have shown that some *ortho*-aminophenols produce cancer of the urinary bladder. Allen, Boyland, Dukes, Horning and Watson (1957) showed that the two *ortho*-aminophenols, 3-hydroxykynurenine and 3-hydroxyanthranilic acid, induced bladder cancer under the same conditions. Higher concentrations of 3-hydroxyanthranilic acid and kynurenine have been found in the urine of patients with cancer of the bladder when compared with urine of either normal subjects or of those with cancer localized in organs other than the bladder (Boyland and Williams, 1955). Perhaps this indicates that a block at the level of the 3-hydroxyanthranilic oxidase (with subsequent accumulation of 3-hydroxyanthranilic acid and of 3-hydroxykynurenine) is the basis of tumours of the bladder.

Dunning, Curtis and Mann (1956) in a study of the relationship between diet and production of cancer by 2-acetylaminofluorene found that cancer of the bladder occurred in almost all of the rats (strain Fisher 344) fed with a diet containing tryptophan (1·4–4·3 per cent). The large amounts of tryptophan necessary for the development of cancer suggest that two *ortho*-aminophenolic derivatives with carcinogenic activity, 3-hydroxyanthranilic acid and 3-hydroxykynurenine might be excreted. If 2-acetylaminofluorene caused a biochemical block in the transformation of these two compounds, it would explain the carcinogenic action in the bladder of this compound. Quagliariello and his colleagues (1958) have concluded that rats treated with 2-acetylamino-fluorene have a block at the level of 3-hydroxyanthranilic oxidase in the liver. In the present work the modification of the tryptophan-nicotinic acid metabolism by treatment with nicotinamide and vitamin $B_6$ in people with tumours of the bladder has been investigated.

### EXPERIMENTAL PROCEDURE

Twenty-four hour specimens of urine were acidified with acetic acid (1 : 1) and centrifuged. Charcoal deactivated with 4 per cent by weight of stearic acid was added to the supernatant and the mixture shaken occasionally for about 5 minutes. The charcoal was filtered off on a small Buchner funnel and washed with water to remove the remaining salts, urea, sugars and aliphatic amino acids. It was then eluted with phenol solution and water. The eluate was evaporated on a water-bath under reduced pressure. To ensure complete removal of the phenol more water was added and the mixture again concentrated. The concentrate thus obtained was applied directly to paper chromatograms.

For routine use about 10 to 20 ml. of urine was suitable, but there was no difficulty in using as little as 5 ml. The amounts of charcoal and phenol used varied because of variations in the metabolite content of the urine, but a suitable amount could easily be estimated after some experience had been gained. If sufficient charcoal had been used the urine filtrate after the initial adsorption was almost or quite colourless.

For routine examinations one-dimensional descending chromatograms developed with butanol : acetic acid : water (4 : 1 : 5, v/v/v) were most suitable. Chromatograms run under such conditions are adequate for almost all purposes provided reference standards are run simultaneously. $R_F$ values can be modified amongst other things, by a metabolite being displaced by other substances in a crowded region of the chromatogram, and by variations in the pH of the applied solution.

*Fluorescence under ultra-violet light.*—When examined in ultra-violet light which has passed through a Wood's glass filter many metabolites are revealed as characteristic fluorescent spots. Ultra-violet light is also the most convenient way of revealing the solvent front. Normal urine contains traces of many unidentified fluorescent substances, some of which may be of plant origin (from the diet) rather than true metabolites.

*Ninhydrin.*—Ninhydrin is of value chiefly for the aromatic amino acids and also, using a modified reagent and the resulting ultra-violet fluorescence, for tryptamine derivatives.

*Ehrlich's reagent.*—This is of particular value for the detection of indole derivatives, pyrrole derivatives and aromatic amines. It is convenient to have two reagents available, differing in acid strength, which will be referred to as the " normal " and " strong " Ehrlich's reagent respectively. The " normal " reagent

TABLE I.—*Chromatographic Evidence of Tryptophan Metabolites in Urine of Patients with Cancer of the Bladder*

| Case number | Kynurenine | 3-hydroxy-kynurenine | 3-hydroxy-anthranilic acid | Anthranilic acid |
|---|---|---|---|---|
| 1. D. E. | +++ | + | +++ | – |
| 2. A. L. | ++ | – | ++ | + |
| 3. G. S. | +++ | – | ++ | + |
| 4. R. A. | – | – | + | + |
| 5. R. G. | ++ | – | ++ | + |
| 6. C. A. | ++ | – | + | – |
| 7. D. M. | ++ | – | ++ | + |
| 8. C. E. | – | – | ++ | – |
| 9. Q. F. | + | – | ++ | + |
| 10. C. S. | – | – | + | – |
| 11. T. P. | – | – | + | + |
| 12. T. C. | + | – | – | + |
| 13. P. L. | + | – | – | – |

+++ = constant presence and large quantities; ++ = constant presence and small quantities; + = not always present or only in small quantities; – = never present.

TABLE II.—*Chromatographic Evidence of Tryptophan Metabolites in Urine of Patients with Cancer of the Bladder, Before and After Treatment with Nicotinamide* (400 *mg.*/pro die *for* 15 *days*)

| Case number | Before nicotinamide treatment | | | | After 15 days of nicotinamide treatment | | | |
|---|---|---|---|---|---|---|---|---|
| | Kynure-nine | 3-hydroxy-kynure-nine | 3-hydroxy-anthra-nilic acid | Anthra-nilic acid | Kynure-nine | 3-hydroxy-kynure-nine | 3-hydroxy-anthra-nilic acid | Anthra-nilic acid |
| 14. T. M. | + | – | +++ | ++ | + | – | – | – |
| 15. M. G. G. | ++ | – | +++ | – | – | – | – | – |
| 16. C. A. | ++ | – | +++ | + | + | – | – | – |
| 17. B. F. | ++ | – | +++ | + | + | – | + | + |
| 18. C. D. | ++ | – | ++ | + | ++ | – | – | – |
| 19. A. S. | + | – | ++ | – | – | – | – | – |
| 20. D. C. | – | – | ++ | + | – | – | ++ | + |
| 21. B. P. | – | – | +++ | + | – | – | + | + |
| 22. T. V. | – | – | +++ | ++ | – | – | – | – |
| 23. G. M. | + | – | +++ | + | + | – | – | + |
| 24. L. S. | ++ | – | ++ | – | + | – | – | – |
| 25. P. L. | +++ | – | +++ | + | – | – | – | – |
| 26. G. R. | ++ | – | ++ | + | ++ | – | ++ | – |
| 27. D. D. E. | – | – | +++ | – | – | – | – | – |
| 28. A. L. | – | – | + | + | – | – | + | ++ |
| 29. G. S. | – | – | ++ | + | – | – | ++ | ++ |
| 30. G. G. | +++ | – | ++ | – | ++ | – | ++ | – |
| 31. D'A. M. | + | – | +++ | + | + | – | – | – |
| 32. S. E. | +++ | – | ++ | – | ++ | – | – | – |
| 33. C. M. | – | – | + | + | – | – | + | + |
| 34. C. D. | ++ | – | +++ | + | – | – | – | – |
| 35. B. S. | + | – | ++ | – | + | – | – | – |
| 36. P. V. | +++ | – | ++ | + | ++ | – | ++ | ++ |

+++ = constant presence and large quantities; ++ = constant presence and small quantities; + = not always present or only in small quantities; – = never present.

TABLE III.—*Chromatographic Evidence of Tryptophan Metabolites in the Urine of Patients with Cancer of the Bladder, Before and After Treatment with Vitamin* $B_6$ (400 *mg.*/pro die *for* 15 *days*)

| | Before vitamin $B_6$ treatment | | | | After 15 days of vitamin $B_6$ treatment | | | |
|---|---|---|---|---|---|---|---|---|
| Case number | Kynurenine | 3-hydroxy-kynurenine | 3-hydroxy-anthranilic acid | Anthranilic acid | Kynurenine | 3-hydroxy-kynurenine | 3-hydroxy-anthranilic acid | Anthranilic acid |
| 37. E. A. | +++ | +++ | + | − | − | ++ | + | − |
| 38. D. G. | − | ++ | + | + | − | − | − | − |
| 39. N. L. | + | ++ | + | − | + | + | + | − |
| 40. D. S. | + | +++ | − | − | + | +++ | − | − |

+++ = constant presence and large quantities ; ++ = constant presence and small quantities ; + = not always present or only in small quantities ; − = never present.

TABLE IV.—*Chromatographic Evidence of Tryptophan Metabolites in the Urine of Patients with Cancer of the Bladder, Before and After Treatment with Nicotinamide and vitamin* $B_6$ (200 *mg.* + 200 *mg.*/pro die *for* 15 *days*)

| | Before vitamin $B_6$ treatment | | | | After 15 days of nicotinamide and vitamin $B_6$ treatment | | | |
|---|---|---|---|---|---|---|---|---|
| Case number | Kynurenine | 3-hydroxy-kynurenine | 3-hydroxy-anthranilic acid | Anthranilic acid | Kynurenine | 3-hydroxy-kynurenine | 3-hydroxy-anthranilic acid | Anthranilic acid |
| 41. P. M. | ++ | ++ | +++ | + | ++ | + | ++ | − |
| 42. C. M. | +++ | − | +++ | − | + | − | + | − |
| 43. S. A. | ++ | + | +++ | ++ | + | − | − | + |
| 44. A. G. | ++ | ++ | ++ | + | + | + | + | − |
| 45. D'A. V. | + | + | + | + | − | − | − | − |
| 46. D. G. | + | ++ | +++ | + | ++ | − | + | − |
| 47. S. M. | + | + | + | ++ | + | − | − | − |

+++ = constant presence and large quantities ; ++ = constant presence and small quantities ; + = not always present or only in small quantities ; − = never present.

is a 2 per cent (w/v) solution of *p*-dimethylaminobenzaldehyde in approximately 1.3N–HCl. The " strong " reagent is a 2 per cent (w/v) solution of the aldehyde in approximately 6 N–HCl. After spraying the papers are allowed to dry at room temperature. With the " normal " reagent, aromatic amines give a yellow colour immediately, whereas colours from indole derivatives may take some time to form. At the pH of the " strong " reagent derivatives of aromatic amines are formed but remain colourless, whereas indole derivatives including indican, give a colour rapidly. It is thus possible to distinguish the two types of compounds even if present in the same spot. Ultimately the picture obtained with both reagents is the same.

*Ammoniacal silver nitrate.*—The papers are sprayed and allowed to dry at room temperature. The reagent is of special value in detecting substances containing readily oxidizable groups such as *o*-aminophenol or catechol derivatives. These rapidly give black or brown-black spots. Many other substances will react, but more slowly.

Urine from 47 patients with cancer of the bladder was collected and analysed every other day for 20 days. At the end of this time 23 of these patients were given 400 mg. per day of nicotinamide (Nicotinamide " Lepetit ") by parenteral injection for 15 days. Four other patients were injected with 400 mg. Vitamin $B_6$ (Benadon " Roche ") per day for 15 days. Seven patients were treated with both vitamin $B_6$ (200 mg.) and nicotinamide (200 mg.). In some patients the studies were continued after surgical removal of the tumours.

### RESULTS

In the urine from 44 of the patients 3-hydroxyanthranilic acid was present ; kynurenine and anthranilic acid were present in 35 and 31 of these cases respectively. 3-Hydroxykynurenine was found in only 11 patients. Additional metabolites such as acetylkynurenine, anthranilic acid glucuronide, kynurenic acid and xanthurenic acid were occasionally present.

In 3 patients 3-hydroxyanthranilic acid was never detected but only kynurenine and some of its derivatives.

In 14 of the 23 patients treated with nicotinamide there was a gradual disappearance of the 3-hydroxyanthranilic acid often with a complete change in the chromatographic pattern. In 2 patients the nicotinamide treatment, begun only after surgical operation, caused the excretion of the metabolites of the tryptophan-nicotinic acid group to return to normal.

Vitamin $B_6$ was given to four patients whose urine contained large amounts of 3-hydroxykynurenine. Normal functioning of the excretory system was obtained only in one case. Another 7 patients were treated with both nicotinamide and vitamine $B_6$, but normal excretion was obtained in only 3 cases, probably because only a half-dose of vitamin $B_6$ and nicotinamide was administered.

(1) Of the three patients who never excreted 3-hydroxyanthranilic acid, biopsies showed that two did not have cancer of the bladder.

(2) All 47 of the cases investigated were followed for 20 days. In some patients the presence of 3-hydroxyanthranilic acid in the urine was always detectable ; in others the excretion of this metabolite was not constant.

(3) In recent studies by others (Benassi and Perissinotto, 1959, 1960) 3-hydroxyanthranilic acid has not been found, although a large number of cases were examined.

(4) In 13 of the subjects examined we have followed the urinary excretion of the metabolites of tryptophan even after surgical removal of the tumour and we have not found that the excretory products have returned to normal. This is important, because it indicates a dangerous and permanent metabolic block.

(5) The nicotinamide treatment appears to cause normal metabolism of tryptophan. This treatment was suggested by our previous researches which indicated the probable role of DPN in the 3-hydroxyanthranilic acid oxidation.

### DISCUSSION

From our results it is evident that in different diseased conditions the normal mechanisms for degradation of tryptophan are altered and that some metabolites of the tryptophan-nicotinic acid group accumulate in the urine. However, with paper chromatography it is not always possible to demonstrate a pattern which differs according to the disease. We feel, however, that there is a characteristic change in the excretory products of people afflicted with cancer of the bladder.

In agreement with our earlier studies (Fedele, *et al.*, 1959, 1961 ; Quagliariello *et al.*, 1958) the researches of Boyland and Williams (1955), and experiments of Tompsett (1959), the results show that 3-hydroxyanthranilic acid is usually present in the urine along with other metabolites of the tryptophan-nicotinic acid group —kynurenine and anthranilic acid—in bladder cancer patients.

After our studies and those of others we have cited, we cannot consider research regarding the correlation between blocks in the tryptophan nicotinic acid metabolism and cancer of the bladder as finished. However, it appears more evident that there is a relationship between metabolites of the tryptophan and cancer of the bladder.

SUMMARY

Urine from patients with bladder cancer usually contains 3-hydroxyanthranilic acid, kynurenine and anthranilic acid. The excretion of 3-hydroxyanthranilic acid is reduced by treatment with large doses of nicotinamide.

### REFERENCES

Allen, M. J., Boyland, E., Dukes, C. E., Horning, E. S. and Watson, J. G.—(1957) *Brit. J. Cancer*, **11**, 212.
Auricchio, S., Quagliariello, E., Rubino, A. and Vecchione, L.—(1959) *Bioch. applic.*, **3**, 113.
Benassi, C. A. and Perissinotto, B.—(1959) ‘ Res. V Jornadas Bioquimicas Latinas,’ Barcelona (Mayo), p. 84.—(1960) *Farmaco*, **15**, 323.
Bonser, G., Clayson, D. B., Jull, I. W. and Pirah, L. N.—(1952) *Brit. J. Cancer*, **6**, 412.
Boyland, E. and Williams, D. C.—(1955) *Biochem. J.*, **60**, V.
Dunning, W. F., Curtis, M. R. and Mann, B. D.—(1956) *Cancer Res.*, **10**, 454.
Fedele, L., Tancredi, F. and Riccardi, I.—(1959) *Atti Soc. it. Urol.*, **32**, (in press).—(1961) *Minerva urol.*, **13**, 1.
Quagliariello, E., Auricchio, S., Casale, M. and Tancredi, F.—(1958) *Boll. Soc. ital. Biol. sper.*, **34**, 970.
Tompsett, S. C.—(1959) *Clin. chim. Acta*, **4**, 411.